# JAK/STAT signaling in liver disease: a therapeutic target or a context-dependent double-edged sword?

**DOI:** 10.1186/s12967-026-08135-9

**Published:** 2026-04-15

**Authors:** Tuokai Wang, Lanjuan Li, Juan Lu

**Affiliations:** 1 State Key Laboratory for Diagnosis and Treatment of Infectious Diseases, National Clinical Research Center for Infectious Diseases, China-Singapore Belt and Road Joint Laboratory on Infection Research and Drug Development, National Medical Center for Infectious Diseases, Collaborative Innovation Center for Diagnosis and Treatment of Infectious Diseases, The First Affiliated Hospital, Zhejiang University School of Medicine, Hangzhou, Zhejiang China; 2 Yuhang Institute of Medical Science Innovation and Transformation, Hangzhou, Zhejiang China

**Keywords:** JAK/STAT signaling pathway, Liver disease, Targeted therapy, Context-dependent

## Abstract

**Background:**

Liver disease remains a major global health burden, and effective targeted therapies are still lacking for many conditions, particularly chronic inflammatory, fibrotic, and malignant liver diseases. The JAK/STAT signaling pathway has emerged as a central regulator of cytokine signaling in the liver and is increasingly considered a potential therapeutic target. However, its biological effects are highly context-dependent.

**Main body:**

In this review, we address a central question: is the JAK/STAT pathway a practical therapeutic target in liver disease, or does its context-dependent biology limit direct clinical translation? We summarize how JAK/STAT signaling regulates antiviral defense, immune tolerance, macrophage polarization, hepatic stellate cell activation, fibrogenesis, and tumor-promoting inflammation across viral hepatitis, autoimmune liver disease, NAFLD/NASH, liver fibrosis, cirrhosis, and hepatocellular carcinoma. Importantly, the evidence indicates that JAK/STAT signaling is not uniformly pathogenic. Its effects vary according to disease stage, upstream cytokine milieu, canonical versus non-canonical activation, and, critically, cell type-specific responses in hepatocytes, immune cells, hepatic stellate cells, and tumor-associated cells.

**Conclusion:**

This review examines the molecular mechanisms of this signaling pathway in various liver pathologies, summarizes the current research into regulating these signals as a potential treatment for chronic liver disease. Rather than broad pathway inhibition, future strategies should focus on cell-specific, disease-stage-specific, and function-selective modulation to suppress pathogenic inflammation and fibrosis while preserving antiviral immunity and tissue-repair programs. This framework may better support precision medicine approaches for chronic liver disease and liver cancer.

## Introduction

About 2 million people worldwide die of liver diseases every year, 1 million of them die of liver cirrhosis-related complications, and another 1 million die of viral hepatitis and hepatocellular carcinoma [1]. In terms of disability adjusted life years (DALYs), liver disease ranks 15th in the global disease burden. In addition to the clinical impact, these diseases also bring huge economic pressure to the global health system [2, 3]. In 2016, the medical expenditure related to chronic liver disease exceeded 32 billion US dollars [4]. In view of the heavy burden caused by liver disease, it is very important to understand its pathogenesis and develop targeted therapy and drug research. Janus kinase/signal transducer and activator of transcription pathway is an evolutionarily conserved signal transduction system, which is involved in many key physiological processes such as hematopoiesis, differentiation, metabolism, and immune regulation [5–8]. The study of this pathway originated from the early exploration of interferon-mediated transcriptional activation. Through continuous scientific exploration, scholars have gradually revealed the structural components and pathogenesis of this pathway [9–11]. Subsequent studies found that there are more than 50 different cytokines in the JAK/STAT network, including interferon (IFNs), interleukin (ILs) and various growth factors [12–14]. Because cytokines play a fundamental role in both humoral and cellular immunity, the JAK/STAT cascade is of great significance in regulating the liver immune microenvironment. This regulatory function is particularly prominent in autoimmune liver diseases such as primary biliary cholangitis (PBC). Experimental evidence showed that Th1-like cell populations in the liver showed a specific JAK/STAT activation mode, which was consistently verified in human PBC cases and corresponding mouse models. The high correlation between these special cells and liver pathological progress highlights the key role of this signaling pathway in the development of liver diseases [15]. In other liver diseases, except PBC, there is an urgent clinical demand for nonalcoholic fatty liver disease (NAFLD) and its progressive nonalcoholic steatohepatitis (NASH), which is mainly due to the lack of approved targeted drugs [16, 17]. So far, lifestyle intervention is still the main prevention and management strategy, but in the late stage of the disease, the curative effect is limited, and patient compliance is poor [18]. Therefore, clarifying the pathogenesis and discovering new therapeutic targets are the core tasks of current research, and inflammation has been recognized as one of the key drivers [16]. Previous studies have widely confirmed that the JAK/STAT signaling pathway is the core hub of cytokine signaling and have identified it as an important therapeutic target for immune-mediated diseases [19]. Scholars have also confirmed that liver inflammation plays a central role in the progression of NASH to liver fibrosis and cirrhosis [16, 20, 21], which makes targeting the JAK/STAT pathway a new approach to treating these chronic liver diseases. However, transforming this pathway from a theoretical target to an effective clinical treatment still faces major unmet clinical needs and complex scientific challenges. Therefore, the key question is not simply whether JAK/STAT signaling is involved in liver disease, but whether it can be therapeutically targeted in a clinically meaningful and biologically safe manner. Accumulating evidence suggests that JAK/STAT signaling exerts divergent effects across different liver diseases and even among different hepatic cell populations within the same disease context. In some settings, it amplifies inflammation, fibrogenesis, immune escape, and tumor progression; in others, it contributes to antiviral defense, immune regulation, or tissue repair. This review examines how the therapeutic relevance of JAK/STAT signaling is shaped by disease context, cell-type specificity, and pathway cross-talk, and we discuss whether future interventions should move beyond broad inhibition toward precision modulation.

## Literature search strategy

We searched PubMed, Embase, Web of Science and the Cochrane Library for studies published from 1 January 2000 to 31 December 2024 (last update 1 March 2025) using the terms (“JAK*” OR “STAT*”) AND (liver OR hepatic OR hepatocellular). To ensure historical continuity, we included landmark studies published before 2000 that elucidated the fundamental biological mechanisms of JAK/STAT, as well as a small number of non-liver-related papers that were critical for clarify ing the pathway architecture. Eligible literature consisted of peer-reviewed original English-language articles or authoritative reviews that explored the role of JAK/STAT signaling in the pathophysiology or treatment of liver disease; single-case reports and studies unrelated to liver disease were excluded. The most relevant references were extensively cited in this narrative review. Given the descriptive scope of this article, no quantitative meta-analysis or formal risk of bias assessment was conducted.

**Fig. 1:**
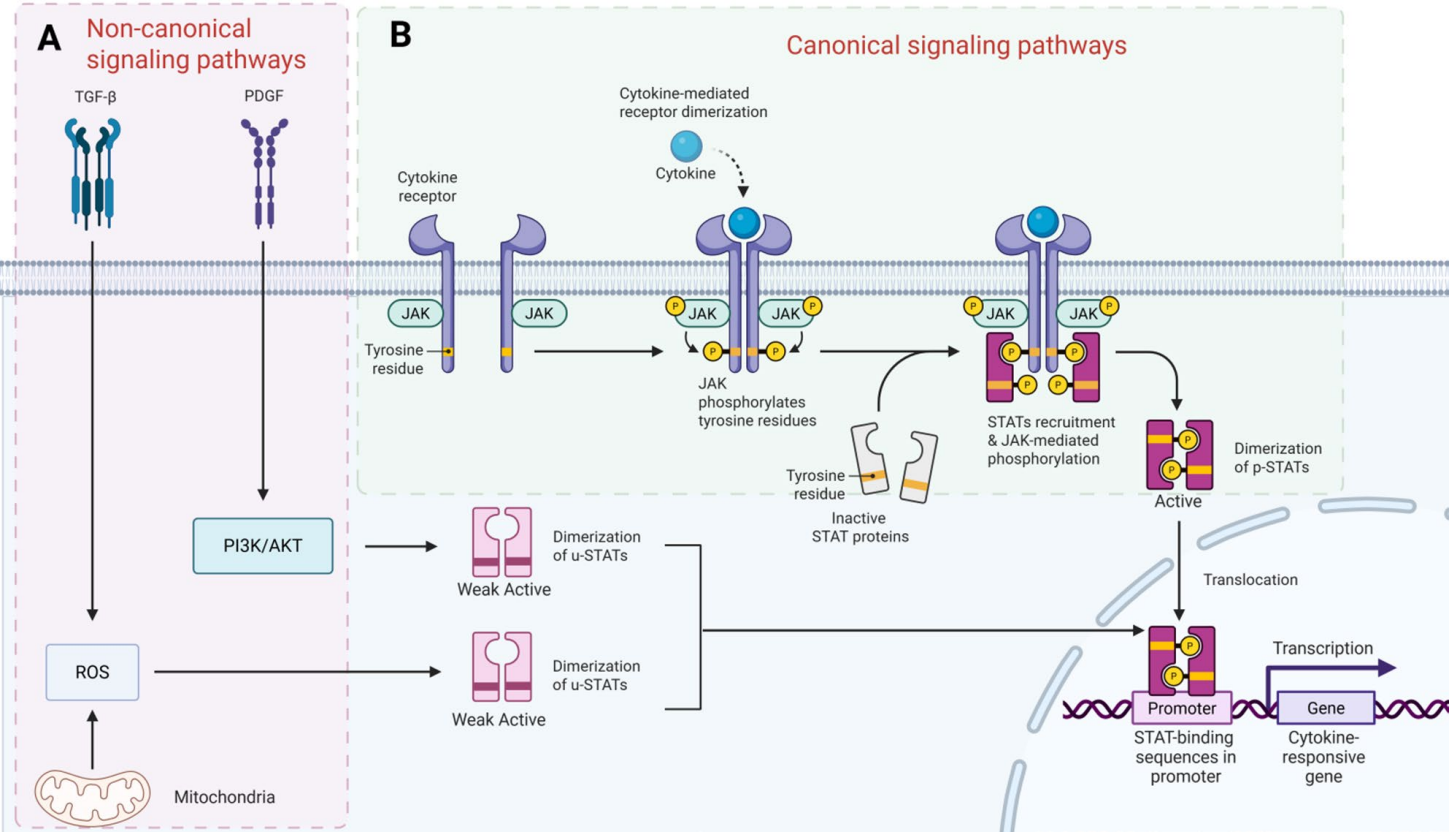
Schematic Diagram of the Canonical and Non-canonical Pathways of the JAK/STAT Signaling Pathway. **(A)** Non-canonical signaling pathways: TGF-β, PDGF, or mitochondrial ROS induce weak activation and dimerization of unphosphorylated STAT (u-STAT) via the PI3K/AKT pathway, independent of the classical cytokine-receptor-JAK cascade. **(B)** Canonical signaling pathway: Cytokines drive receptor dimerization, JAKs phosphorylate receptors and STATs to form active p-STAT dimers. These p-STAT dimers translocate to the nucleus, bind to promoters, and regulate the transcription of cytokine response genes. (Created in biorender.com)

## Core molecules and regulatory mechanisms of the JAK/STAT pathway

JAK family includes JAK1, JAK2, JAK3 and TYK2, and its structural characteristics are the C-terminal tyrosine kinase domain (TKD) and adjacent pseudokinase domain (PKD) **(**Table 1**)** [22–25]. Members of the JAK family are receptor-proximal, non-receptor tyrosine kinases that couple cytokine-receptor engagement to downstream signaling [26–31]. The signal transducer and activator of transcription (STAT) family includes STAT1-STAT4, 5 A, 5B and STAT6 [32–35], and each member has an amino-terminal protein interaction domain, a central DNA binding domain, an SH2 domain and a carboxyl-terminal transcriptional activation domain [36, 37].

**Table 1:** Core Molecules of the JAK/STAT Pathway and Their Roles in Liver Disease Pathogenesis

Molecular Category	Core Molecule	Function Description	Associated Diseases	Ref.
JAK Kinases	JAK1	Acts as a receptor-associated non-receptor tyrosine kinase	AIH, HCC	[26, 27]
	JAK2	Regulates cytokine signaling and immune response, contributing to fibrosis and immune modulation	Liver fibrosis, HCC, cirrhosis	[28, 29]
	JAK3	Involved in T-cell signaling, regulating immune response and tolerance	AIH	[30]
	TYK2	Regulates cytokine signaling, particularly in immune responses and inflammation	Hepatitis, immune-related diseases	[31]
STAT Transcription Factors	STAT1	Participates in antiviral immune response, immune cell differentiation, and immune evasion	HBV, HCV, liver fibrosis	[22, 23]
	STAT2	Key in IFN signaling, particularly in antiviral responses	HBV, HCV	[24, 25]
	STAT3	STAT3 shows marked cell-context specificity: (1) Hepatocytes: drive acute-phase gene expression and promote survival/regeneration; (2) Kupffer/monocyte-derived macrophages: provide feedback anti-inflammation acutely but, when chronically activated, sustain IL-6-dependent, tumor-promoting inflammation; (3) Hepatic stellate cells: induce α-SMA and ECM genes, accelerating fibrogenesis; (4) HCC cells: maintain cancer-stem-cell traits, immune evasion and angiogenesis.development	Acute liver injury, NASH, fibrosis, HCC	[20, 55], [99, 109]
	STAT5A/B	Participates in hematopoiesis, immune response, liver cell proliferation, and immune modulation in liver diseases	AIH, liver fibrosis, cirrhosis	[36, 37]
	STAT6	Primarily regulates Th2 immune responses, immune tolerance, and fibrosis, influencing liver immune responses	PSC	[38, 39]

JAK protein is characterized by four major domains: FERM domain, which is a complex composed of protein 4.1, ezrin, radixin and moesin, Src homology 2 (SH2) domain, pseudokinase domain and kinase domain [36, 38–40]. Structurally, the FERM domain presents a clover like conformation and is composed of three subdomains, F1, F2 and F3 [41]. The main function of the FERM domain and SH2 domain is to promote the binding between the JAK protein and its receptor. At the same time, pseudokinase domain is responsible for regulating the catalytic activity of the kinase domain. The kinase activity is crucial for the phosphorylation of tyrosine residues on the receptor, which will initiate a phosphorylation cascade involving downstream signaling molecules [42]. In summary, these four major domains can be further divided into seven independent regions, namely JAK homology (JH) domains 1 to 7 [23, 43, 44]. The JAK/STAT signaling pathway has two distinct activation modes: canonical and non-canonical pathways (Fig. 1). As the earliest discovered and most deeply studied pathway, the canonical pathways are characterized by signal transduction strictly depending on the sequential phosphorylation and activation of JAK and STAT proteins [45–48]. In this pathway, cytokines or growth factors bind to cell surface receptors, activate JAKs, and then phosphorylate STAT (p-STAT) proteins. The p-STAT protein forms a dimer and is transported to the nucleus to directly regulate the transcription of target genes, such as interferon-stimulated genes ISGS [49]. The canonical signaling pathway is still the mainstream focus of current research, which is usually activated by cytokines, hormones, or growth factors. For example, interferon type I (IFNs), interleukin-6 (IL-6), prolactin, growth hormone (GH), and a variety of pro-inflammatory/pro-fibrosis cytokines all play a role through this pathway [45, 49–51]. The specific mechanism is: the ligand and receptor bind to activate JAK kinase, which p-STAT proteins (such as STAT1, STAT2, STAT3, and STAT5), and the p-STAT protein is transported to the nucleus to regulate gene expression [48, 49, 52, 53]. This classic model plays a central role in many physiological and pathological processes, including immune response, cell proliferation, differentiation, and apoptosis, and has become a therapeutic target for cancer, autoimmune diseases, viral infection, and other diseases [49–51, 53]. The canonical pathway has become a key target for drug research and development because it plays a central role in a variety of diseases. At present, several JAK inhibitors (such as ruxolitinib and baricitinib) have entered the clinical trial stage **(**Table 2**)**. At the same time, the research on non-canonical pathways is accelerating-this pathway not only reveals the complexity of the cellular signaling network and compensation mechanism, but also explains why the effect of inhibiting classical pathways is limited in specific situations [48]. Non-canonical signaling pathways, also known as “non-classical” pathways, refers to pathways that do not rely on JAK-mediated STAT tyrosine phosphorylation cascades, but instead utilize unphosphorylated STAT (u-STAT), the nuclear/mitochondrial functions of JAK2, or alternative modifications of STAT by other kinases to regulate gene expression in a parallel or complementary manner, They are critical to the realization of complete biological effects, such as ISG transcription or mRNA translation. These pathways do not follow the classical receptor-JAK-STAT tyrosine phosphorylation cascade, but still converge on STAT-dependent or JAK-associated functions [48]. Studies have shown that in addition to the classical interferon signal, some growth factors, such as PDGF and TGF-β1, can activate the JAK/STAT signal through canonical or non-canonical pathways, highlighting the universality of the non-canonical mode [54]. Montero and other researchers also pointed out that in interstitial lung disease, a variety of growth factors can activate the JAK/STAT signaling pathway through canonical and non-canonical dual pathways. This indicates that the non-canonical activation mode not only exists in immune signal transduction, but also occurs in pathological processes such as fibrosis [51]. In view of the key role of fibrosis in the occurrence and development of liver diseases, further exploration of this mechanism is expected to provide innovative strategies for the diagnosis and treatment of liver diseases.

**Table 2:** Representative JAK inhibitors mentioned in this review

Drug	Selectivity	Key evidence relevant to liver disease in this review	Major safety risks	Approved indications	Ref.
Ruxolitinib	JAK1/JAK2	In murine autoimmune cholangitis, ruxolitinib reduced portal inflammation, bile duct injury, pro-inflammatory cytokine transcription, and T-cell accumulation, while promoting Treg expansion and macrophage M2 polarization; No approved liver-specific indication at present	Infections, herpes zoster, anemia, thrombocytopenia	Myelofibrosis, polycythemia vera, graft-versus-host disease	[23, 59]
Baricitinib	JAK1/JAK2	Mentioned in this review as a representative clinically used JAK inhibitor. However, no approved liver-specific indication at present	Infections, herpes zoster, thrombosis, lipid abnormalities	Rheumatoid arthritis; also approved/authorized in some regions for alopecia areata, atopic dermatitis, and COVID-19	[23, 49]
Peficitinib (ASP015K)	Pan-JAK, with relative preference for JAK3/JAK1	Included as an example of clinically used JAK inhibition in autoimmune disease. No approved liver-specific indication at present	Infections, cytopenia, liver enzyme abnormalities	Rheumatoid arthritis (approved in some countries/regions)	[75, 76]

Note: This table summarizes representative JAK-targeted drugs explicitly mentioned in the manuscript or cited references. For several agents, inclusion reflects translational relevance rather than established liver-specific efficacy. At present, no JAK inhibitor has been approved specifically for liver disease

## Viral hepatitis

### Interferon signaling and regulation of antiviral immune responses

In viral hepatitis, the JAK/STAT pathway, as the core mechanism of IFN signal transduction, regulates the antiviral immune response of the host. When type I interferon (such as IFN-α/β) activates this pathway, the downstream STAT protein is phosphorylated and transported to the nucleus, thereby inducing the expression of interferon-stimulated genes (ISGs) such as RIG-I and IFIT3. These genes can directly inhibit viral replication [55–57]. The latest research reveals that the JAK/STAT pathway plays a dual role in antiviral immunity: on the one hand, it plays a key role in virus clearance through the IFN-ISG axis [58]; on the other hand, some viruses will hijack this pathway to enhance their own replication. For example, inhibition of the JAK/STAT pathway will reduce ISG expression and promote virus accumulation in host cells [57, 59]. In addition, many viruses (such as HIV, SARS-CoV-2, and HCV) escape immune clearance by destroying this pathway. Its strategies include degradation of STAT protein, inhibition of phosphorylation and upregulation of SOCS expression, which eventually lead to chronic infection [60–62].

### Hepatitis B

Previous studies have shown that persistent inflammation in chronic viral hepatitis is closely related to the abnormal activation of the JAK/STAT pathway. In the process of hepatitis B virus (HBV) infection, sustained viral antigen stimulation can induce sustained activation of STAT3. Recent studies have further strengthened this mechanistic link, suggesting that STAT3 activation is an important contributor to HBV-induced liver inflammation [63]. In the animal model, the expression of p-STAT3 in the liver tissue of three-month-old HBV transgenic mice was significantly increased (*p* < 0.05), and was positively correlated with the expression of inflammatory genes (Saa1/2, S100a8/9/11, IL1β, etc.). Clinical cohort analysis showed that the number of p-STAT3 positive hepatocytes in young patients with chronic HBV was significantly positively correlated with serum ALT level (*r* = 0.7977, *P* = 0.0100), and the number of p-STAT3 positive cells in patients with ALT > 40 U/l was significantly higher than that in patients with normal ALT (*p* < 0.05). Together, these findings indicate that STAT3 activation, as reflected by phosphorylated STAT3, is positively associated with the severity of liver inflammation. Such activation not only drives the release of pro-inflammatory factors such as IL-6, but also promotes the infiltration of inflammatory monocytes and macrophages into liver tissue, thereby aggravating tissue damage [64]. Jinglin Tang et al. Confirmed by STAT3 inhibition experiment (using Stattic): inhibition of STAT3 activation can reduce the expression of liver inflammatory genes, monocyte/macrophage infiltration, and the severity of overall inflammation in HBV transgenic mice. These preclinical findings suggest that STAT3 inhibition may alleviate liver inflammation and reduce inflammatory mediator expression in experimental HBV models [64]. In addition, JAK/STAT pathway dysfunction is associated with impaired immune tolerance. In the process of chronic infection, the virus can weaken the antiviral effect of interferon by inhibiting this pathway, while maintaining pro-inflammatory signal transduction, thus forming a chronic inflammatory cycle [58]. Recent experimental work suggests that JAK1 activity may facilitate HDV replication in specific model systems, raising the possibility of a context-dependent proviral role [65]. However, because JAK1 also mediates antiviral interferon signaling, its net role in HBV/HDV co-infection remains incompletely defined and should not yet be considered definitive across different biological settings.

### Hepatitis C

In addition to outlining the role of the JAK/STAT pathway in hepatitis B-related liver disease, Tuck et al. Found that STAT3 played a key role in the impaired T-cell immune response during hepatitis C virus infection. This study confirmed that the binding of extracellular HCV core protein with gC1qR could further activate the PI3K/AKT pathway. This pathway then activates STAT3 in human monocytes, macrophages, and dendritic cells (DCs) through the IL-6 autocrine pathway. This process drives antigen-presenting cells (APCs) to produce abnormal inflammatory responses, which provides a new explanation for the HCV immune escape mechanism [66]. Scholars further explored the role and mechanism of STAT3-regulated long non-coding RNA (lncRNA) in hepatitis C virus (HCV) replication. Previous studies have shown that the host phosphatidylinositol 4 kinase and its product PI4P are involved in HCV replication through the effector bovine sterol binding protein [67, 68]. The researchers established a stable cell line with STAT3 overexpression, and screened four lncRNAs (lnc-IGF2-AS, lnc-7sk, lnc-SChLAP1, and lnc-sra1) up-regulated by STAT3 through lncRNA PCR chip. The experiment revealed that lnc-IGF2-AS and lnc-7SK could promote HCV replication by regulating the expression of phosphatidylinositol 4-phosphate.This finding is consistent with previous experimental observations and supports a role for STAT3-regulated lncRNAs in HCV replication, thereby suggesting a potential avenue for future mechanistic and therapeutic investigation. These findings suggest that JAK/STAT inhibition may reduce inflammatory damage, but may also compromise antiviral defense, highlighting a therapeutic trade-off.

## Autoimmune liver disease

### Autoimmune hepatitis

Autoimmune liver diseases, such as autoimmune hepatitis (AIH), primary biliary cholangitis (PBC), and primary sclerosing cholangitis (PSC), are closely related to excessive immune activation caused by the destruction of peripheral self-tolerance [69–71]. Abnormal signal transduction of the JAK/STAT pathway can activate pro-inflammatory cytokine signals, destroy the immune balance, and promote the development of autoimmune diseases [72, 73]. In AIH research, more and more evidence shows that the JAK/STAT pathway plays a central role in mediating liver inflammation, immune cell infiltration, and hepatocyte apoptosis [74]. Although JAK inhibitors for rheumatoid arthritis, systemic lupus erythematosus, and other autoimmune diseases have entered clinical application [75, 76], targeted research in the field of AIH is still continuing to advance, aiming to find more effective molecular targets and treatment strategies. The role of the JAK/STAT pathway in the pathogenesis of AIH is mainly reflected in the regulation of T cell differentiation and hepatocyte injury. In terms of immune regulation, this pathway promotes the polarization of Th1 and Th17 cells by activating the JAK2/JAK3/STAT1/STAT3 signaling pathway. This immune imbalance leads to the release of TNF-α, IFN-γ, IL-2, IL-17, and other inflammatory factors, which further aggravate liver injury [74]. In addition to immune regulation, this pathway is also directly involved in hepatocyte apoptosis. In the experimental model, Rhoifolin (ROF) inhibits hepatocyte apoptosis by reducing the level of apoptosis-related proteins (Cleaved-Caspase-3/9, Bax), upregulating anti-apoptotic protein Bcl-2, and inhibiting IL-6/JAK2/STAT1/STAT3/BNIP3 pathway [74]. This experiment confirmed the potential of ROF as a natural product in the treatment of AIH, provided an experimental basis for the development of new drugs for AIH, and provided a new idea for the research of related targets.

### Primary biliary cholangitis

PBC is characterized by autoimmune destruction of small bile ducts driven by CD8^+^ T cells [77]. Early studies have shown that Th1 cells participate in bile duct injury by secreting interferon-γ (IFN-γ), which aggravates the inflammatory response in the portal region [78, 79]. In view of JAK1 and JAK2 as key downstream checkpoints for interferon signaling, subsequent experiments compared the liver tissue of the control group with that of the chronic IFN-γ overexpression mouse model (ARE-Del). The results showed that the expression levels of JAK1, JAK2, TYK2 and STAT1 were significantly increased in the experimental group [80]. In summary, these findings emphasize the key role of IFN-γ in promoting the pathogenesis of primary biliary cholangitis by regulating immune cell activation and differentiation through the JAK/STAT pathway [81–83]. At the treatment intervention level, researchers used the ARE-Del model to evaluate the efficacy and mechanism of JAK1/2 inhibitor ruxolitinib in the treatment of autoimmune cholangitis. At the immunological level, ruxolitinib could not only inhibit the transcription of pro-inflammatory genes such as IFN-γ and IL-6, but also reduce the number of CD4^+^ and CD8^+^ T cells in the spleen and liver. At the same time, the drug enhances the number of regulatory T cells (Tregs) through the STAT6-IRF4 pathway and promotes the transformation of macrophages from M1 to M2 phenotype, providing a new perspective for immune-mediated bile duct injury. In addition, this study confirmed that ruxolitinib treatment significantly improved portal vein inflammation and small bile duct injury in ARE-Del+/- mice, thereby reducing liver pathological score [59]. This targeted therapy for JAK/STAT signaling pathway may provide a promising alternative strategy for patients with poor response to conventional metabolic therapy.

### Primary sclerosing cholangitis

PSC is a chronic liver disease, including cholestasis, characterized by inflammation and bile duct scarring. The exact pathogenesis is still unclear, and the current medical practice lacks effective treatment options [84, 85]. In biliary diseases such as PSC and PBC, the main pathological feature is bile fibrosis, which is usually consistent with cholestasis and interruption of bile acid regulation [86]. In order to explore how the liver regenerates after injury and responds to inflammation, scientists used the 3,5-diethoxycarbonyl-1,4-dihydrocolidine (DDC) model, which is an experimental substitute for human PSC. The observations in this model emphasize the dynamic changes of signaling pathways. Importantly, these data emphasize the necessity of immunophenotypic transition, especially the transition from M1 to M2 polarization, a phenomenon previously associated with JAK/STAT axis, especially STAT6 [87]. For the autoimmune cholangitis model discussed above, the JAK1/2 inhibitor ruxolitinib has been proven to be very effective. It can significantly inhibit portal vein inflammation and catheter injury, and reduce the frequency of T cells in the liver and spleen. In the mechanism, ruxolitinib inhibited the expression of STAT1 and the release of inflammatory factors such as IL-6, TNF and MCP1 in macrophages, and increased STAT6. This regulatory effect promotes the transition from pro-inflammatory M1 macrophages to reparative M2 phenotypes. In the DDC model, this balance is considered to be essential for liver regeneration [59]. Therefore, future studies may give priority to the clinical trials of these drugs in patients with PSC to verify their safety and the ability to reverse fibrosis, and ultimately to find reliable and effective treatment methods.

**Fig. 2:**
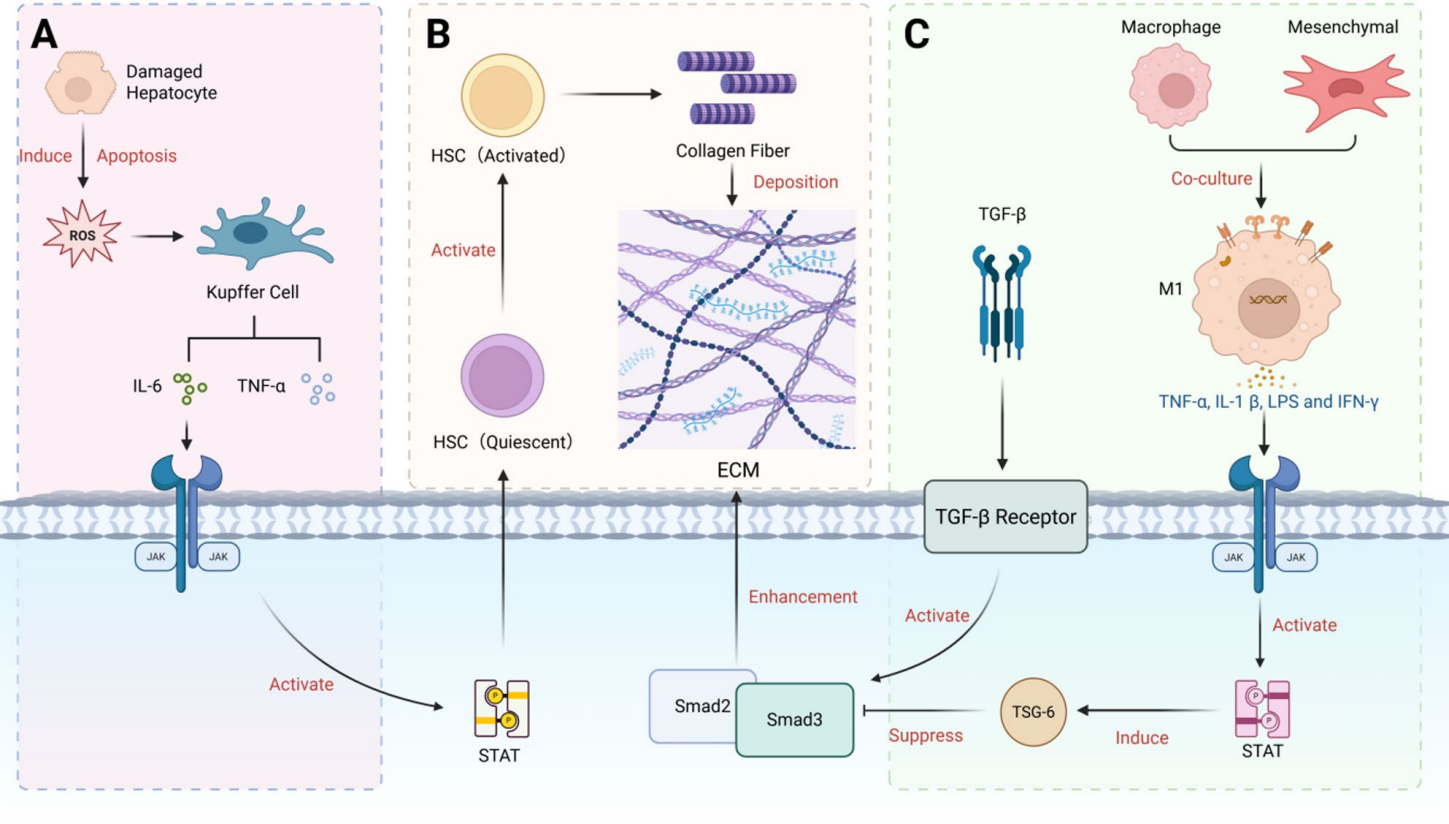
Regulation of Signaling Pathways Associated with Liver Fibrosis. **(A)** Damaged hepatocytes undergo apoptosis, producing ROS that activate Kupffer cells to secrete IL-6 and tumor necrosis TNF-α. These cytokines activate STAT through the JAK pathway. **(B)** Activated STAT promotes the activation of resting HSCs, and activated HSCs secrete collagen fibers deposited in ECM. The Smad pathway further promotes the formation of ECM. **(C)** In a co-culture system of macrophages and mesenchymal stem cells, M1 macrophages secrete inflammatory factors that activate STAT and produce TSG-6, which regulates extracellular matrix (ECM) formation by inhibiting the TGF-β/Smad pathway. (Created in biorender.com)

## Nonalcoholic fatty liver disease and Nonalcoholic steatohepatitis

Nonalcoholic fatty liver disease (NAFLD), from simple fatty liver to steatohepatitis, is an important global health problem leading to liver-related diseases. With the aggravation of overnutrition and obesity, its prevalence continues to rise. Nonalcoholic steatohepatitis (NASH) is the progressive stage of NAFLD. It has the characteristics of fat accumulation, hepatocyte damage and inflammation, and has become one of the main causes of liver cirrhosis and HCC [88–92]. Regarding pro-inflammatory pathogenesis, experiments demonstrate that the JAK/STAT pathway serves as a key signaling axis mediating NK cell-driven NASH progression. Its activation drives NASH development through the release of pro-inflammatory factors and hepatocyte injury [93]. This study suggests that NK cells and the JAK-STAT pathway may serve as potential therapeutic targets for NASH, laying the groundwork for developing novel treatments. While p38γ deficiency reduced lipid accumulation by inhibiting JAK/STAT, and p38γ was up-regulated under fatty acid loading, and promoted lipid accumulation by activating JAK/STAT. Its inhibition can significantly reduce liver injury and steatosis [94], and miR-142-5p can effectively improve the pathological characteristics of NASH by inhibiting TSLP-JAK/STAT signal. At the gene regulation level of NASH, miR-142-5p successfully reduces inflammation and steatosis by targeting TSLP and inhibiting JAK/STAT signal mediated by it [95]. In terms of protection and repair, relevant studies have found that the JAK/STAT signaling pathway mediates the crosstalk between macrophages and hepatocytes through the OSM-STAT3-ARG2 axis, promotes fatty acid oxidation (FAO) of hepatocytes, and thus reduces the steatosis and inflammatory response of NASH. JAK/STAT signaling pathway was significantly up-regulated in mice with myeloid cell-specific overexpression of SPP1 (Spp1KI Mye). This activation is mainly achieved by oncostatin M (OSM) secreted by macrophages. After binding with osmr on the surface of hepatocytes, OSM activates STAT3 phosphorylation (p-STAT3) and then upregulates the expression of arginase 2 (ARG2), indicating that the activation of STAT3 is the key link in the increase of ARG2 expression. In vitro experiments showed that the expression of ARG2 and STAT3 phosphorylation induced by spp1ki mye macrophage conditioned medium was significantly decreased after osmr siRNA knockout of hepatocytes. It is worth noting that in vivo, the injection of OSM-neutralizing antibody can inhibit the STAT3-ARG2 pathway, leading to the pathological aggravation of NASH, confirming the core role of the JAK/STAT-ARG2 axis in reducing liver steatosis [96]. This mechanism provides a potential target for the treatment of Nash, that is, it is hopeful to improve liver metabolism by enhancing the OSM-JAK/STAT-ARG2 signal axis between macrophages and hepatocytes. In addition, the study also found that IL-22 can reduce steatosis and hepatitis by activating the JAK1/STAT3 pathway, regulating inflammation, metabolism and tissue repair-related genes. Therefore, the IL-22-IL-22R axis is regarded as a potential strategy for the treatment of NAFLD, which can improve insulin sensitivity through the JAK1/STAT3 pathway and reduce lipid metabolism disorders [97]. Regarding future treatments for NAFLD/NASH, beyond research into targeted therapies for JAK/STAT-related pathways, a noteworthy new direction involves how to suppress the pro-inflammatory and pathogenic effects of this pathway in disease while promoting protective and reparative actions. This approach aims to steer the regulatory function of the JAK/STAT pathway toward beneficial outcomes. These studies indicate that JAK/STAT signaling is not uniformly pathogenic in NASH, and its therapeutic manipulation requires functional selectivity.

## Liver fibrosis and cirrhosis

### The driving role of hepatocyte activation in ECM Deposition

Chronic hepatocyte injury and persistent inflammation are usually precursors of cirrhosis, which will develop into fibrosis and eventually lead to sclerosis. JAK/STAT signaling cascade has been identified as the key driver of this progress, and also the inducement of subsequent hepatocellular carcinoma (HCC) [98]. After chronic liver injury, the liver usually enters a pathological stage called fibrosis, which is characterized by excessive accumulation of extracellular matrix (ECM) [99]. The activation of hepatic stellate cells (HSCs) is the core of the fibrosis process. After activation, these cells will transdifferentiate into myofibroblasts and then produce excessive ECM. Current evidence suggests that STAT3 is a major pro-fibrotic effector of the JAK/STAT axis in many experimental settings, particularly through promoting HSC activation and fibrosis progression, although this role is context-dependent rather than universal [99, 100]. At present, the research on JAK/STAT signaling pathway in liver fibrosis is mainly divided into two categories: To explore the mechanism of exogenous pathological stress factors activating this pathway, and to develop drug therapy targeted to inhibit this pathway. The literature shows that different pathological stimuli induce HSCs to present a pro-fibrotic phenotype by activating the JAK/STAT signaling pathway. For example, in the context of HIV co-infection and alcohol abuse, HSCs phagocytize apoptotic bodies produced by damaged hepatocytes. This phagocytic event stimulates the synthesis of intracellular reactive oxygen species (ROS) and IL-6, thereby activating the JAK/STAT3 pathway (Fig. 2) [100]. These findings reveal how intercellular signaling is mediated by JAK/STAT to transform hepatocyte injury into a signal of HSC activation. In the field of treatment research and development, a lot of efforts have focused on finding drugs that can inhibit HSC activation by blocking STAT3 signal transduction. For example, natural molecule (-)-catechin-7-o-β-d-apiofuranoside (C7A) has shown a strong ability to inhibit HSC activation, and its main mechanism is to block the STAT3 pathway [99]. By blocking STAT3 signal transduction, C7A can not only reduce the accumulation of ECM but also inhibit the activity of hepatic stellate cells. The anti-fibrosis effect confirmed in cell and animal models provides empirical support for the potential application of this molecule in the management of liver fibrosis.

### Signaling crossover of fibrogenic cytokines

Current studies increasingly regard liver fibrosis as the product of multiple pathways, highlighting the dynamic relationship between IL-6/JAK/STAT-dominated inflammatory environment and TGF-β-driven fibrosis. The hallmark of fibrosis pathology is the extensive cross-regulation between inflammatory mediators such as the JAK/STAT axis, IL-6 and pro-fibrosis cytokines such as TGF-β, which together promote the activation of hepatic stellate cells (HSCs). There was a significant correlation between STAT3 phosphorylation and the activation of TGF-β/Smad signaling cascade. It is worth noting that pro-fibrotic stimuli such as HCV infection or CCl4 exposure often upregulate these two pathways at the same time, and can usually be achieved through drug-synchronous inhibition [101–103]. Studies on JAK2/STAT3 pathway have shown that such blocking can not only inhibit inflammatory signal transduction, but also indirectly regulate TGF-β-induced extracellular matrix deposition. The potential mechanism may involve autophagy regulation or downstream gene transcription changes [103]. In addition, this signal interaction is not a strict one-way promotion process, but involves a complex feedback loop. Evidence shows that JAK/STAT activation can induce the production of anti-inflammatory protein TSG-6 to alleviate fibrosis in specific situations, such as macrophage and mesenchymal stem cell co-culture systems. The mechanism is that the inflammatory factors released by M1 macrophages (including TNF-α, IL-1 β, LPS and IFN-γ) activate the JAK/STAT1/3 pathway and promote macrophages and ASCs to synergistically upregulate TSG-6 expression. Elevated TSG-6 then inhibits the TGF-β/smad3 signaling pathway, thereby inhibiting the expression of α-SMA, the key marker of hepatic stellate cell fibrosis, and finally produces an anti-fibrosis effect [104]. These findings highlight the dual characteristics of the JAK/STAT pathway in the fibrotic liver microenvironment: it not only acts as a transmitter of inflammatory signals such as IL-6, but also regulates the TGF-β signaling pathway through specific mediators.

### Hepatitis-to-liver fibrosis progression

The JAK/STAT signaling pathway plays a key role as a bridge in the pathological progression of hepatitis to liver fibrosis, mainly by regulating the inflammatory response and activating hepatic stellate cells (HSCs) to promote the process of fibrosis. In order to further verify the regulatory mechanism and function of this pathway in the transformation from hepatitis to liver fibrosis, researchers carried out multi-level research. The animal model study provides an empirical basis for the anti-fibrosis effect of the JAK/STAT pathway on liver fibrosis and lays a foundation for clinical transformation. CCl1 secreted by macrophages binds to the CCR8 receptor on the surface of HSC and activates the JAK/STAT signaling pathway in CCl4 induced liver fibrosis mouse model. This process can promote HSC activation, inhibit cell apoptosis and accelerate the process of liver fibrosis. This discovery reveals the new mechanism of CCl1-CCR8-JAK/STAT pathway regulating hepatic stellate cell activation and.

apoptosis, and provides a new idea for the targeted treatment of liver fibrosis [105]. In another experiment of this induction model, researchers first confirmed that GATA4 mediated the reversal of adult liver fibrosis through inactivated HSC, revealing a new regulatory mechanism of GATA4 directly inhibiting EPAS1/HIF2α. At the same time, they confirmed the fibrogenic effect of HIF2α in the activation of HSC [106]. GATA4 is a key regulatory factor for the inactivation of adult HSC and the reversal of fibrosis. This study found that GATA4 deletion can induce HSC activation and fibrosis progression, and its overexpression can directly inhibit EPAS1/HIF2α pathway, downregulate fibrosis-related genes and upregulate anti-fibrosis genes, so as to promote hepatic stellate cell inactivation and reverse fibrosis. In addition, the stabilization of HIF2α can also induce fibrosis [106]. Future research can explore targeted regulation of this pathway as a new therapeutic strategy. In fibrosis, STAT3-centered signaling appears more consistently pro-fibrotic, making this axis relatively more attractive for intervention.

**Fig. 3:**
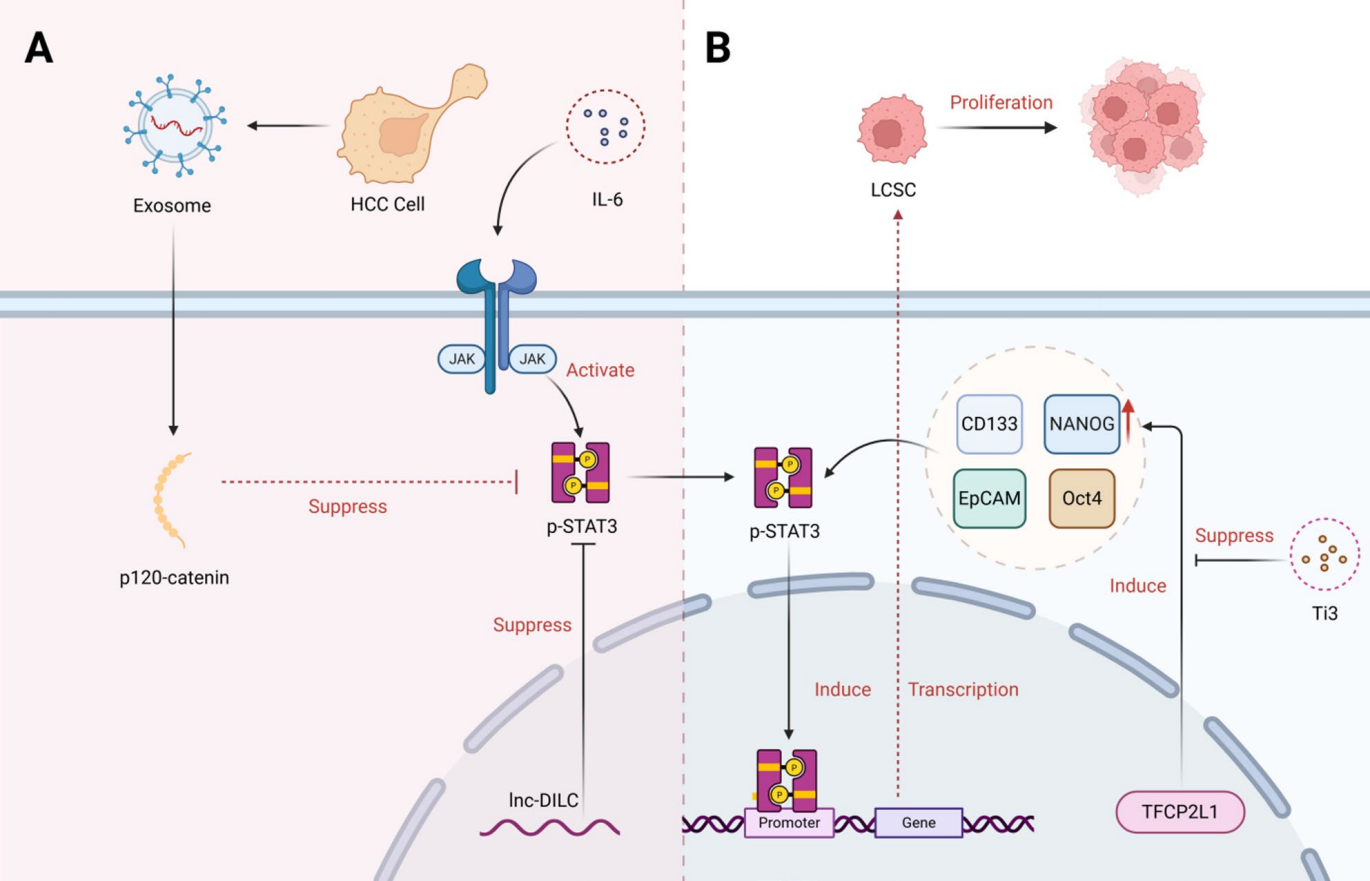
STAT3 regulates the stemness of liver cancer stem cells. **(A)** Liver cancer cells first produce large amounts of exosomes. Exosomes can mediate the joint suppression of p-STAT3 by p120-catenin and lnc-DILC, which activates the IL-6/JAK pathway. p-STAT3 enters the nucleus to regulate gene transcription, ultimately promoting LCSC proliferation. **(B)** p-STAT3 induces TFCP2L1 to upregulate stemness markers such as CD133 and NANOG, thereby promoting LCSC proliferation. The small molecule Ti3 can inhibit this process. (Created in biorender.com)

## Hepatocellular carcinoma

### The development and evolution of hepatocellular carcinoma

A prominent feature of hepatocellular carcinoma (HCC) is the disorder of the JAK/STAT signaling cascade. Evidence shows that this signal axis promotes a variety of tumor occurrence behaviors, such as cell proliferation, survival, neovascularization and metastasis [107–109]. This persistent signal transduction is often triggered by upstream growth factors and cytokines (such as IL-17 and IL-6), especially in the microenvironment of cirrhosis and chronic inflammation [109–111]. In addition, this pathway is closely related to metabolic iron imbalance. For example, excessive iron accumulation combined with reduced hepatidin levels can accelerate the pathogenesis of HCC through JAK/STAT [112]. From the perspective of mechanism, the complex protein gene interaction network regulates the signaling pathway. The cytokine signaling suppressor (SOCS) family (especially SOCS1 and SOCS3) is the key brake of JAK/STAT activity, and its silencing or reduction will lead to continuous activation of the pathway [113–115]. For example, EYA2, a tumor suppressor, drives SOCS3 transcription by forming a complex with Dach1, thereby inhibiting JAK/STAT signaling and blocking HCC progression. However, an EYA2 mutation will destroy this inhibitory mechanism [113]. On the contrary, specific immune checkpoint proteins (such as HHLA2) and long-chain non-coding RNA (such as LINC00346) have been confirmed to activate the JAK/STAT pathway, thereby promoting immune escape, anti-apoptotic ability and cell proliferation of HCC [116, 117].

### The association between hepatocellular carcinoma stem cell maintenance and STAT3

Signal transduction and sustained activation of STAT3 are key drivers of cancer stem cells (CSCs) in hepatocellular carcinoma (HCC) [118]. Studies have shown that phosphorylation and activation of STAT3 can directly promote the self-renewal, proliferation and metastasis of liver cancer stem cells (LCSCs) (Fig. 3) [119, 120]. In vivo and in vitro model experiments, targeted protein degradation technology for STAT3 has been proven to effectively inhibit the growth of LCSCs [118]. It is worth noting that the upregulation of the STAT3 pathway is significantly positively correlated with the expression of dry markers such as CD133, EpCAM, Oct4 and NANOG [121–123]. Meanwhile, TFCP2L1 plays an important role in maintaining the pluripotency of embryonic stem cells. Clinical studies have found that TFCP2L1 is highly expressed in HCC tissues and is associated with shorter overall survival and disease-free survival, which is confirmed as an independent prognostic factor and an important evaluation index. The model further revealed that TFCP2L1 could promote CSC dryness and enhance sorafenib resistance by upregulating Nanog to activate the JAK/STAT3 pathway. In addition, animal model screening found that small molecule Ti3 targeting the active domain of TFCP2L1, combined with sorafenib, significantly improved the sensitivity of HCC cells to treatment, suggesting that TFCP2L1 can support further evaluation as a potential target for HCC treatment and provides a new combined treatment strategy for overcoming drug resistance [122]. In addition, the researchers found that p120-catenin secreted by HCC cells can inhibit the STAT3 pathway, while lnc-DILC exerts tumor suppressor gene function by inactivating IL-6/STAT3 signaling pathway. These two mechanisms can inhibit the expansion of liver cancer stem cells and lay the foundation for a treatment strategy based on microenvironment regulation and exogenous intervention.

### Microenvironmental inflammation-tumor transformation mechanisms

Existing researchs have shown that a variety of cytokines in the liver cancer microenvironment not only directly affect the fate of tumor cells, but also profoundly reshape the immune microenvironment by activating the JAK/STAT cascade (Table 3) [98, 124]. However, the highly complex functional role of this pathway in different cell types and pathological backgrounds has brought major challenges to clinical transformation [125]. Understanding how JAK/STAT mediates the transformation of inflammation to tumorigenesis is crucial to the development of new targeted therapeutic strategies. IL-6 family cytokines are considered to be the key mediators connecting inflammation and the development of hepatocellular carcinoma. These cytokines are secreted by infiltrating inflammatory cells in the process of chronic liver injury and activate the JAK/STAT pathway through the gp130 receptor subunit. In terms of mechanism, IL-6 family cytokines activate JAK kinase after binding with receptors, and the latter promotes its dimerization and transport to the nucleus to regulate gene expression through phosphorylation of STAT transcription factors (mainly STAT3) [124]. This process represents an important mechanism linking chronic inflammation to HCC development [98]. It is worth noting that oncostatin M (OSM), a member of the IL-6 family, plays an important role in the HCC microenvironment. It not only participates in inflammatory response, but also promotes epithelial-mesenchymal transition (EMT), obtains cancer stem cell characteristics and develops metastatic phenotypes [124]. IL-27 receptor (IL-27R) signal also shows an unexpected tumor-promoting effect in the HCC microenvironment. Researchs have found that the IL-27R signal is a new immune checkpoint for regulating NK cell activity. IL-27R deficiency can enhance the aggregation and activation of cytotoxic NK cells in liver injury and tumor tissues, thus inhibiting the progression of HCC. This indicates that the JAK/STAT-related cytokine signaling may simultaneously inhibit the anti-tumor immune response in the microenvironment [126]. In addition to exogenous cytokine stimulation, the continuous activation of the JAK/STAT pathway is also closely related to gene mutation. Studies have revealed that JAK1 and STAT3 are high-frequency mutation genes related to the JAK/STAT pathway in HCC. This study further confirmed that JAK/STAT pathway activation is closely related to M2 polarization of tumor-associated macrophages (TAMs), which usually promotes tumor growth. In HCC tumors with JAK/STAT pathway mutations (such as STAT3 and ep300 mutations), significantly increased M2 macrophage infiltration was observed, accompanied by the upregulation of PD-L1 expression in these macrophages. This indicates that pathway mutations reshape the immune microenvironment and lead to poor prognosis [127]. Therefore, the JAK/STAT signaling pathway, as the core driver of inflammation to tumor transformation in the HCC microenvironment, is continuously activated through the response to IL-6 family cytokines, mutation-driven activation and the absence of a negative feedback regulation mechanism. The activation of this pathway not only directly promotes the malignant phenotype of tumor cells but also reshapes the immune microenvironment by inducing M2 macrophage polarization and inhibiting NK cell function. These findings provide a theoretical basis for future targeted therapy, gene therapy and immune microenvironment regulation. In HCC, persistent STAT3 activation is closely linked to stemness and immune evasion, supporting its role as a more actionable target in advanced disease.

**Table 3:** Context-dependent functions and therapeutic implications of JAK/STAT signaling across liver diseases

Disease context	Dominant cell types involved	Predominant JAK/STAT function	Therapeutic implication	Key caution/limitation	Ref.
Viral hepatitis (HBV/HCV)	Hepatocytes, monocytes/macrophages, dendritic cells	Bidirectional: STAT1/2-mediated IFN signaling supports antiviral defense, whereas persistent STAT3 activation may amplify inflammatory injury and viral persistence	Broad inhibition is unlikely to be ideal; selective modulation may reduce inflammation while preserving antiviral immunity	Excessive pathway inhibition may impair ISG induction and weaken virus control	[55–66]
AIH	T cells, hepatocytes	Predominantly pro-inflammatory, promoting Th1/Th17 polarization and hepatocyte injury	JAK inhibition may help restore immune balance in immune-mediated hepatitis	Immunosuppression and infection risk remain concerns	[72–76]
PBC/PSC	T cells, macrophages, cholangiocyte-associated immune microenvironment	Predominantly immune-activating, but macrophage reprogramming suggests some context-specific reparative potential	JAK1/2 inhibition may reduce biliary inflammation and immune-mediated injury	Evidence is still largely preclinical; disease heterogeneity remains important	[59, 77–87]
NAFLD/NASH	Hepatocytes, macrophages, NK cells	Context-dependent: can promote inflammation and steatosis, but may also support metabolic adaptation and tissue repair through OSM/STAT3 or IL-22/JAK1/STAT3 signaling	Functional selectivity is required rather than broad inhibition	Suppressing protective STAT3 programs may worsen steatosis or impair repair	[93–97]
Liver fibrosis/cirrhosis	Hepatic stellate cells, macrophages, injured hepatocytes	Predominantly pro-fibrotic, especially via STAT3-driven HSC activation and ECM deposition	STAT3-centered intervention may be more therapeutically attractive in fibrosis than in earlier inflammatory disease	Crosstalk with TGF-β/Smad and non-canonical signaling may limit single-pathway blockade	[99–106]
HCC	Tumor cells, cancer stem-like cells, TAMs, NK cells	Predominantly tumor-promoting, supporting stemness, immune evasion, angiogenesis, and drug resistance, especially through persistent STAT3 activation	STAT3 appears to be a more actionable target in advanced HCC	Effects on antitumor immunity and combination strategies require careful evaluation	[109, 118–127]

## Conclusion

Liver disease remains a major global health burden. According to the data of the global disease burden project, more than 2 million people died from major liver diseases such as acute hepatitis, cirrhosis and liver cancer in 2010, accounting for about 4% of the total number of deaths in the world that year [128, 129]. Therefore, the study of liver disease is of great significance. As discussed above, JAK/STAT signaling exerts important but context-dependent functions across multiple liver diseases, including viral hepatitis, autoimmune liver disease, fibrosis/cirrhosis, and hepatocellular carcinoma, with effects varying according to disease stage, cytokine milieu, and cell type. As the core hub of cytokine signaling, the JAK/STAT pathway regulates immune response, inflammatory process, hepatic stellate cell activation and fibrosis progression. In addition, by mediating IFN signal response, the JAK/STAT pathway plays a central role in antiviral immunity during viral hepatitis, and provides a mechanism for virus immune escape. In autoimmune liver disease, a number of studies have confirmed that JAK/STAT pathway imbalance can aggravate the destruction of immune tolerance, promote abnormal activation of immune cells and liver inflammation. In the pathophysiology of liver fibrosis and cirrhosis, the JAK/STAT pathway not only promotes the progression of fibrosis by regulating the activation of hepatic stellate cells and the deposition of extracellular matrix, but also aggravates the vicious circle of fibrosis through the interaction with TGF-β/Smad signaling pathway. In hepatocellular carcinoma, JAK/STAT signaling not only maintains the survival of tumor stem cells but also drives tumor progression through immune escape and drug resistance mechanisms. Can JAK/STAT signaling be considered a practical therapeutic target for liver diseases? We believe the answer depends on the disease context, cellular compartment, and the biological characteristics of a specific stage. Although sustained JAK/STAT activation typically promotes inflammation, fibrosis, immune evasion, and tumor progression, in other circumstances, the same pathway may also maintain antiviral defense, metabolic adaptation, or tissue repair. Therefore, the therapeutic significance of JAK/STAT signaling lies not in broad inhibition, but in understanding its context-dependent functions and leveraging them through precise regulation. At present, however, much of the supporting evidence remains preclinical, and liver-specific clinical validation is still limited.

## Challenge and Outlooks

Although the JAK/STAT pathway has great potential as a therapeutic target for a variety of liver diseases, its clinical transformation still faces multiple challenges. Firstly, the dual characteristics of this pathway make its therapeutic effect complex and changeable in different pathological situations. Although a large number of animal model experiments have been successful, these models can not fully reproduce the complexity and heterogeneity of human liver disease. For example, inhibition of this pathway in viral hepatitis may weaken the antiviral immune response of the host, while it may help to restore the immune balance in autoimmune liver disease. Therefore, how to accurately regulate the JAK/STAT pathway in different disease states and avoid adverse side effects will become the core direction of future research. Secondly, the non-canonical signaling pathway and the cross-regulation between the JAK/STAT and other pathways (such as TGF-β) add complexity to targeted therapy, and it is urgent to develop more elaborate treatment strategies, especially for specific cell types such as hepatic stellate cells or tumor-associated macrophages [101–103]. With the deepening understanding of the liver tumor microenvironment, future research should focus on the role of the JAK/STAT pathway in immune escape and tumor immune surveillance, and explore its potential for combined treatment with immune checkpoint inhibitors. JAK/STAT signaling pathway provides broad prospects for targeted treatment of liver diseases. However, because this pathway is involved in a variety of physiological functions, systemic inhibition may trigger off-target effects such as immunosuppression [109, 130]. The development of tissue or cell-specific targeted drugs is a key challenge to be solved in the future. Liver diseases are highly heterogeneous, and there may be differences in pathological mechanisms between different etiologies and individuals [130, 131]. This brings challenges to the development of universal treatment programs, and future research needs to focus more on precision medicine and personalized treatment strategies.

## Data Availability

Not applicable.

## Abbreviations

JAK: Janus kinase
STAT: Signal transducer and activator of transcription
HCC: Hepatocellular carcinoma
DALYs: Disability adjusted life years
PBC: Primary biliary cholangitis
NAFLD: Nonalcoholic fatty liver disease
NASH: Nonalcoholic steatohepatitis
TKD: Tyrosine kinase domain
PKD: Pseudokinase domain
JH: JAK homology
FERM: Protein 4.1, ezrin, radixin and moesin
SH2: Src homology 2
ISGs: Interferon-stimulated genes
IFNs: Interferons
ILs: Interleukins
GH: Growth hormone
PDGF: Platelet-derived growth factor
TGF-β: Transforming growth factor-beta
HBV: Hepatitis B virus
ALT: Alanine aminotransferase
HCV: Hepatitis C virus
HDV: Hepatitis D virus
DCs: Dendritic cells
APCs: Antigen-presenting cells
lncRNA: Long non-coding RNA
AIH: Autoimmune hepatitis
PSC: Primary sclerosing cholangitis
TNF-α: Tumor necrosis factor-alpha
IFN-γ: Interferon-gamma
ROF: Rhoifolin
DDC: 3,5-diethoxycarbonyl-1,4-dihydrocolidine
FAO: Fatty acid oxidation
OSM: Oncostatin M
ECM: Extracellular matrix
HSCs: Hepatic stellate cells
ROS: Reactive oxygen species
C7A: (-)-catechin-7-O-beta-D-apiofuranoside
CSCs: Cancer stem cells
LCSCs: Liver cancer stem cells
OSM: Oncostatin M
EMT: Epithelial-mesenchymal transition
SOCS: Suppressor of cytokine signaling
TAMs: Tumor-associated macrophages
